# Patient, family member and caregiver engagement in shaping policy for primary health care teams in three Canadian Provinces

**DOI:** 10.1111/hex.13516

**Published:** 2022-06-15

**Authors:** Peter Hirschkorn, Ashmita Rai, Simone Parniak, Caillie Pritchard, Judy Birdsell, Stephanie Montesanti, Sharon Johnston, Catherine Donnelly, Nelly D. Oelke

**Affiliations:** ^1^ School of Nursing University of British Columbia, Okanagan Kelowna British Columbia Canada; ^2^ School of Rehabilitation Therapy Queen's University Kingston Ontario Canada; ^3^ School of Public Health University of Alberta Edmonton Alberta Canada; ^4^ Imagine Citizens Network Calgary Alberta Canada; ^5^ Department of Family Medicine University of Ottawa, Bruyére Research Institute and Institut du Savoir Montfort Ottawa Ontario Canada; ^6^ Rural Coordination Centre of British Columbia Vancouver British Columbia Canada

**Keywords:** collaborative practice, integration, interprofessional teams, patient engagement in policy, primary care, primary health care, team‐based care

## Abstract

**Introduction:**

Improving health services integration through primary health care (PHC) teams for patients with chronic conditions is essential to address their complex health needs and facilitate better health outcomes. The objective of this study was to explore if and how patients, family members, and caregivers were engaged or wanted to be engaged in developing, implementing and evaluating health policies related to PHC teams. This patient‐oriented research was carried out in three provinces across Canada: British Columbia, Alberta and Ontario.

**Methods:**

A total of 29 semi‐structured interviews with patients were conducted across the three provinces and data were analysed using thematic analysis.

**Results:**

Three key themes were identified: motivation for policy engagement, experiences with policy engagement and barriers to engagement in policy. The majority of participants in the study wanted to be engaged in policy processes and advocate for integrated care through PHC teams. Barriers to patient engagement in policy, such as lack of opportunities for engagement, power imbalances, tokenism, lack of accessibility of engagement opportunities and experiences of racism and discrimination were also identified.

**Conclusion:**

This study increases the understanding of patient, family member, and caregiver engagement in policy related to PHC team integration and the barriers that currently exist in this engagement process. This information can be used to guide decision‐makers on how to improve the delivery of integrated health services through PHC teams and enhance patient, family member, and caregiver engagement in PHC policy.

**Patient or Public Contribution:**

We would like to acknowledge the contributions of our patient partners, Brenda Jagroop and Judy Birdsell, who assisted with developing and pilot testing the interview guide. Judy Birdsell also assisted with the preparation of this manuscript. This study also engaged patients, family members, and caregivers to share their experiences with engagement in PHC policy.

## 1 INTRODUCTION

Patient engagement has evolved over time and is implemented in different areas within the health care system.^1^ Patient engagement is defined as the process of health care professionals, health care organizations, and provincial policymakers collaborating with patients, family members, and caregivers to improve the quality of care provided by the health system.^2^ This collaborative process allows patients, family members, and caregivers to influence decisions that will affect the care they receive.^2^ Patient engagement is known to have positive effects, such as reducing emergency department readmissions and improving patient satisfaction,^2^ which can reduce health costs and improve population health.^3^ Engaging patients in health care changes the philosophy of health care from providing care *to* patients to providing care *with* patients,^3^ while considering a diverse number of perspectives related to the health care system.^4^ Canadian organizations, such as health authority patient advisory groups and patient partner organizations (e.g., Patient Voices Network, IMAGINE Citizens Network), aim to provide opportunities for patients to be engaged in policy and practices to improve health care.^3^ However, despite increased interest and commitment to patient engagement, patient and family engagement in policy at the organizational and system level is an area that continues to require improvement in Canada.^5^

Patient engagement is crucial to improving primary health care (PHC) delivery and the health system as a whole.^6^ Roles and expectations of patients, knowledge and attitudes of patients or health professionals, transparency, communication, organizational support, power dynamics, patient representation, and training are some of the barriers and enablers to patient engagement identified in the literature.^7^ Similarly, equality and diversity are some of the overlooked areas in patient engagement.^7^ Current approaches to patient engagement include patient surveys, townhalls, patients as representatives or quality improvement partners in patient advisory councils and resources that support patient engagement initiatives.^7^, ^8^ In addition, the evidence in the literature is more focused on patient engagement at the individual care planning level rather than policy levels.^8^ A cross‐case PHC policy analysis conducted by Lukey et al.^9^ found that although PHC policies in British Columbia (BC), Alberta (AB), Ontario (ON) and Quebec (QC) have some focus on patient engagement in policy; overall, there is little focus on this topic with no clear information on patient engagement in policy development, implementation and evaluation. Therefore, further research in this area is required to gather evidence on patient engagement in policy processes.^10^ For the purpose of this study, a policy is defined as guiding principles or courses of action at provincial and regional levels (e.g., policies that guide interprofessional PHC service delivery). This study consists of three phases: Phase I (policy analysis of provincial and regional policy), Phase II (interviews and deliberative dialogues) and Phase III (knowledge translation and development of final recommendations). This paper only focuses on the interview aspect of Phase II of the study. The Phase II interviews aimed to understand if and how patients, family members and caregivers in three Canadian provinces (BC, AB, ON) were engaged or wanted to be engaged in the development, implementation and evaluation of policy for PHC teams integration.

### 1.1 Context

PHC teams vary between the three provinces where the interviews took place. Primary care networks (PCNs) are a model for delivering interprofessional PHC services and were established in AB in 2003.^11^ There are currently 41 PCNs serving 3.8 million patients in AB.^12^ In 2018, PCNs were also introduced in BC with the goal of increasing patients' access to primary care providers^9^ by utilizing interprofessional team‐based care.^13^ In contrast, Ontario Health Teams (OHTs) were established in 2019 and are integrated care delivery systems utilized to deliver primary care services in ON.^14^ OHTs replaced the former Local Health Integration Networks (LHINs) in ON.^15^ One of the aims of this change was to facilitate the integration of health services to be performed by representatives of those who are actually served by the OHTs.^15^

Provincial government health agencies in BC, AB and ON all encourage the engagement of patients in policy development, implementation and evaluation to improve the quality of health care;^2^, ^9^, ^16^, ^17^ however, there is significant variation in how patients are engaged in policy development in these provinces.^9^ For example, patients may be included as representatives on PCN boards in AB, whereas patient, family and caregiver engagement were prioritized as an important element in codesigning the new OHTs.^9^ Furthermore, a cross‐case analysis of provincial PHC team policy found that although BC, AB and ON emphasize the importance of patient engagement in policy development, many policy documents from these provinces do not provide further details on the methods of patient engagement.^9^ This cross‐case analysis also found that ON and AB were more advanced with patient engagement in policy development when compared to BC.^9^

## 2 MATERIALS AND METHODS

### 2.1 Research design

We used interpretive description for this study,^18^ a qualitative methodology that requires a clear practice goal and an understanding of what is lacking and what is known in the existing literature.^18^ This approach allows researchers to generate logical questions from background information and helps one to develop new insights while considering the context of previous knowledge.^18^ The cross‐case policy analysis in Phase I identified policies for PHC teams integration from the three provinces and provided context for the data collection and analysis in Phase II. Interpretive description was a suitable method for this study, given the objectives of the study were to understand patient, family member, and caregiver engagement in PHC policy processes and how patients wanted to be further engaged in PHC team policy.

This patient‐oriented research study also included two patient partners on the research team. The research team collaborated with patient partners to develop the interview guide (Appendix SA) and the patient partners conducted pilot interviews to test the interview questions. The interview questions were then revised based on the feedback from patient partners.

The study was approved by the appropriate institutional ethics boards in each province, while the Behavioural Research Ethics Board at the University of British Columbia, Okanagan was the Board of record. The ethics number is H18‐02130.

### 2.2 Sampling and recruitment

A quota sampling strategy that integrated the guidelines of purposive sampling^18^ was used to obtain a diverse sample of 9–10 participants in each province. The interview phase took place at the beginning of the COVID‐19 pandemic creating unique challenges for participant recruitment; however, the research team made efforts to recruit participants with diverse demographic characteristics in geographic location, ethnicity, sex, age, education/employment status and annual household income. This strategy allowed the research team to recruit 29 participants across BC (*n* = 10), AB (*n* = 9) and ON (*n* = 10). Conducting 10 interviews in each province was determined by the research team to be appropriate as interpretive description methodology indicates that this is an appropriate approach provided that one acknowledges the existence of potentially differing perspectives of patients not included in the sample.^18^ AB had one less participant than the desired number. Patients, family members or caregivers of patients who were 19 years and over, living in BC, AB or ON, having two or more chronic conditions and seeing two or more health care providers were included in the study. Individuals who lacked the cognitive capacity to provide informed consent were excluded from the study. Previous history/experience of being engaged in PHC policy was not part of the eligibility criteria for participants, and all interested individuals who met the inclusion and exclusion criteria were encouraged to take part in the study regardless of their experience in PHC policy activities. Patients were recruited through patient advocacy organizations and/or patient and family advisory networks (e.g., Patient Voices Network [BC], Association of Family Health Teams, LHINs and OHTs patient and caregiver networks [ON], IMAGINE Citizens Network [AB]) as well as through the research team's networks. An email invitation, consent form and demographic questionnaire were sent to all potential participants. The demographic questionnaire was used to screen the participants for their eligibility to take part in the interviews. Email invitations were sent to 11 interested individuals in BC, out of which 10 participants were interviewed as one participant did not respond to the email invitation. In both AB and ON all invited participants took part in the interviews (AB = 9/9, ON = 10/10). Verbal consent was obtained from participants who chose to provide their consent and demographic information orally. Participants had the autonomy to choose if they wanted to complete their interview face to face, via telephone or via an audio/video call. However, only one face‐to‐face interview was conducted before all interviews were completed virtually via Zoom™, Skype for Business™ or by telephone, due to the COVID‐19 pandemic. The recruitment was stopped after the targeted number of participants was achieved.

### 2.3 Data collection

Data were collected via semi‐structured interviews. Interviews took place between February and December of 2020. Interviews with study participants were conducted one‐to‐one by trained research assistants at the participating institutions in each province. The interviewers were not known to the participants. Three of the four interviewers were female and were either graduate students or had a graduate degree in nursing or health sciences (A. R., C. P., S. P.). The fourth interviewer was male and was a science undergraduate student with an interest in health care (P. H.). The interviewers introduced themselves as research assistants working on the project and explained the goals of the project to the participants by using the script in the interview guide (Appendix SA). Each interview lasted approximately 45–60 min where participants were asked about their experiences as a patient, family member or caregiver in developing, implementing, and evaluating policies and structures that support integration in interprofessional PHC teams. The participants were also asked if and how they would like to be engaged in policy activities and potential measures to enhance patient engagement in policy processes. No follow‐up interviews were conducted, but a second interview was completed with a participant in ON to accommodate their schedule.

### 2.4 Data analysis

Interviews were audio‐recorded with participants' consent; field notes were taken when participants were not comfortable being recorded. All interview recordings were transcribed verbatim by a transcriptionist; field notes were transcribed by research assistants. Interview transcripts were not validated by participants. Data were analysed for common themes^19^ using NVivo™ Version 12.0 software with an inductive coding approach. Coding was completed by the same research assistants who conducted the interviews (A. R., C. P., P. H., S. P.). The research assistants in each province used the same inductive coding approach in a single NVivo project. Other members of the research team reviewed the coding approach; the coded data in NVivo for each province were then used to identify common themes, which were outlined in a provincial report. These themes were shared with the other members of the research team who were involved in the analysis (C. D., N. D. O., S. M., S. J., J. B.), and were discussed in a team meeting. As the themes identified in each province's report were reviewed, the themes and information identified in the reports were compared with coded data to ensure they were accurately summarized. A cross‐provincial analysis was also completed using the data coded in each province to identify common themes across the three provinces. It is also worth noting that several of the research team members come from clinical backgrounds in nursing (A. R. and N. D. O.), occupational therapy (C. D.) and medicine (S. J.). These research team members provided valuable insight in considering the results and their previous and current clinical experience. Patient partners were also involved in the interpretation of the interview data. Following these analyses, an evidence synthesis of the interview data was developed for each province. This evidence synthesis formed the foundation for discussions at deliberative dialogues in each province. These will be reported elsewhere.

## 3 RESULTS

### 3.1 Demographics

Demographic information was collected from interview participants in each province (Table 1). Certain demographic factors were only collected in BC and ON and were not collected in AB (Table 1). Nineteen participants identified as female, nine participants identified as male and demographic data was missing for one participant.

**Table 1:** Demographic information collected from BC, AB and ON (*n* = 29)

		Province		
		BC (*n* = 10)	ON (*n* = 10)	AB (*n* = 9)
**Age**				
18–40		<5	<5	<5
40–65		<5	<5	<5
65 And over		<5	<5	<5
**Ethnicity**				
White		8	7	7
East or Southeast Asian		<5	<5	0
South Asian		<5	0	0
African		0	0	<5
Indigenous		0	0	<5
**Population of the place of residence**				
<10,000		<5	<5	0
10,000–49,999		<5	<5	<5
50,000–249,999		<5	<5	0
250,000–999,999		<5	0	<5
1 million+		0	<5	<5
**Marital status**				
Single		<5	<5	Data not available
Married/common‐law		5	6	Data not available
Widowed		<5	1	Data not available
Separated/divorced		<5	0	Data not available
**Educational status**				
No‐postsecondary		0	0	Data not available
Postsecondary		10	9	Data not available
**Employment status**				
Employed full or part‐time		<5	<5	Data not available
Retired		<5	6	Data not available
Receiving provincial disability		<5	<5	Data not available
Unemployed		<5	0	Data not available
**Combined household income**				
<$50,000		<5	<5	Data not available
>$50,000		5	7	Data not available

Abbreviations: AB, Alberta; BC, British Columbia; ON, Ontario.

### 3.2 Key themes

Participants' experiences varied across the three provinces and the interview data yielded three key themes. These themes included motivation for policy engagement, experiences in policy engagement and barriers to engagement in policy. These themes are described in detail below.

#### 3.2.1 Motivation for policy engagement

Interview participants shared their experiences with the care received from PHC teams, which may provide insight into their motivation for being engaged in PHC policy. In all three of the provinces, there was low satisfaction among participants regarding the collaboration between their PHC team members, and many participants expressed that they did not feel as if they were being cared for by a team.

*My individual providers ‐ I really like all of them, but what I have found [is] that a lot of the work with connecting and communicating and organizing falls on me, and so I kind of feel like I'm the one who does all of the administrative work related to my health*. (Participant B001)

Furthermore, the degree to which patients were engaged in their care was provider‐dependent. Some participants felt that their health care providers included them in the decision‐making process related to their care, while others felt that they were excluded from this process and their opinion was not valued or they had to make several efforts to be included as a member of their care team. The majority of participants interviewed in all provinces described the importance of engaging patients, caregivers and family members in patient care to improve health outcomes.

*I think you have to be a participant in it as much as the doctor is, to you know, help them to make the right decisions and choices for you*. (Participant B008)

Most participants were satisfied or somewhat satisfied with the care that they received from their PHC team; however, it was common for participants to be unsatisfied with the care they received from a particular health care provider within their PHC team or their PHC team as a whole. These experiences caused many participants to desire the opportunity to communicate with providers, decision‐makers and policymakers to voice their concerns regarding the changes that they would like to see in PHC policy.

#### 3.2.2 Experiences with policy engagement

More than half of the participants in BC (*n* = 6/10) and almost all the participants in AB (*n* = 7/9) and ON (*n* = 10/10) had some experience in policy processes at local, regional, provincial and national levels. There was diversity in the type of policy engagement that participants were involved in, which included provincial policy committees, patient advisory groups/committees, national networks, reviewing medical school curricula and responding to surveys. The level at which this engagement took place was also diverse with it occurring at regional, provincial, and national levels. It was evident that all the participants in this study were willing to engage or continue their engagement in policy activities to improve health services integration through PHC teams.

*I would be happy to be involved and have my voice heard any opportunity I'm given. I've never turned down an opportunity for giving input since I joined the LHINs [Local Health Integration Networks]*. (Participant O008)

Many participants in all three provinces emphasized that engaging patients and integrating their lived experiences into the development, implementation, and evaluation of PHC policy can contribute to improving the health system.

*I feel like my kind of full‐time job is being a patient. I have a lot of medical needs, a lot of procedures I do at home, and I've spent a lot of time in the hospital so I feel like I have a pretty good pulse on like what is lacking in the system, what patients need, what actually matters to patients, so I'm happy to give that feedback in any way*. (Participant B001)

Participants who were engaged in policy shared the importance of being ‘the voice of the ordinary person’, and bringing ‘a sense of reality to [the] table’, (Participant A004). They felt satisfied, empowered and hopeful when their voices were heard, ‘Well, it's transformative. Right? It's an amazing feeling of hopefulness’, (Participant A008). However, participants also highlighted that there were barriers and facilitators to engagement in PHC policy.

#### 3.2.3 Barriers to engagement in policy

Participants who had experience with engagement in policy development, implementation and evaluation and those without experience, shared barriers to engagement opportunities that they had experienced. The most discussed barriers included lack of opportunities for engagement, power imbalances and tokenism. Additionally, participants also highlighted barriers to policy engagement, such as lack of accessibility to opportunities in policy engagement; patients' health conditions; and racism, sexism and ageism. These barriers are described in the following sections and details on additional barriers identified by participants are included in Table 2.

##### Opportunities for engagement

The results of the interviews in BC showed a lack of opportunity for patients to be engaged with decision‐makers at the policy level, ‘The main challenge is there's no avenue for participation. And there is an extreme lack of communication with the people that should be communicating with patients’ (Participant B009). It was also found that most participants in BC were unaware of the engagement opportunities surrounding policy development, implementation, and evaluation, ‘Patients have to come out and seek out the opportunities involved’ (Participant B001). Some participants in BC, AB and ON were not able to engage in policy activities due to geographical and technological limitations (e.g., lack of transportation, remote location, lack of internet access or technological device).

Experiences with a lack of patient engagement at every step of the decision‐making process were similar across the three provinces. Participants who were engaged in policy activities in BC, AB and ON were unsure if their voices were being heard and/or if their feedback or engagement led to any changes in policy,

*People asked, and I provided information… you never actually see the full circle of what the survey results are*. (Participant A007)

Participants highlighted the need and importance of collaborating with decision‐makers, for example, follow‐up by reviewing the findings with patient partners and closing the loop with patients by sharing the outcomes of their engagement with them.

*So, is my voice being heard? Kind of… Not to a great extent. I think people hear that there's [a] need for intersectoral collaboration and patient engagement, but I think the vehicles to do that aren't being provided*. (Participant O006)

Participants also suggested that connecting patients to their primary care provider at the clinical level via patient satisfaction surveys and other kinds of feedback surveys could provide patients with another avenue for engagement in the primary care system, ‘It's easier to talk to people that are close to you’ (Participant O001). Additionally, participants described that PHC team members could facilitate capacity building through education about potential opportunities for further engagement. For instance, many ON participants had learned of the LHIN Patient and Family Advisory Committees and other engagement opportunities through their family doctors.

##### Token versus genuine patient engagement

Lack of equitable power distribution between health care organizations and patients was frequently mentioned during the interviews in all three provinces. Most participants described their engagement in policy development, implementation and evaluation as being a tokenistic experience with uneven power distribution. For example, patients were invited to be engaged but they were not engaged throughout decision‐making in policy processes,

*We felt like we got a nice pat on the head. Oh, thank you, this is great. And I actually felt like it was almost sometimes you're just checking a box. Let's have a family… a patient… a patient family committee, check!*. (Participant O002)

Both tokenism and power imbalances were most likely to occur when health professionals thought, ‘I'm an expert, and you're not’ (Participant B010). This resulted in a lack of trust in the system and participants felt their opinions were not adequately considered. Overall, participants felt there was a greater need for policymakers to truly listen to individuals with lived experiences and to act upon their concerns, feedback and/or recommendations towards improving PHC teams integration,

*pay attention… People are people and they're not little boxes and don't forget people's humanity*. (Participant A006)

Some participants shared their experiences of being genuinely engaged in policy where their input was valued and considered by the decision‐makers,

*I've also had experiences where they've valued the feedback that we provide, so it's a bit of both, a balance*. (Participant A007)

##### Accessibility of engagement opportunities

Some participants stated that the accessibility of engagement opportunities was a barrier to them. Participants stated that it was difficult to find transportation to meeting locations, work commitments made attending meetings difficult, and that some participants did not have access to a computer or internet, preventing them from participating virtually. Furthermore, for some patients, their health conditions made it difficult to access the engagement opportunities as they were not well enough to participate in commitments that required a lot of time or energy, ‘Right now wouldn't be the time, because I'm dealing with this other medical condition. And so, I'm very much from one day to the next. I don't know how I'm feeling’, (Participant A003). These participants highlighted the need to create engagement opportunities that were more accessible and that did not have as large of a time commitment.

##### Experiences with racism and discrimination

Some participants had unpleasant experiences with engagement in policy, which they connected to their social identity.

*Some of the other projects that I've been a part of, I've been very excited about and I was… you know, I fit the requirements and all that stuff and yet when the conversations are happening, when we are talking about the things that we want to see, the lived experience that would help the project go one direction or another, I get dismissed. Like, I'm not listened to as much as they would to other people and I have a feeling it has something to do with bias. Like, you know, like, I'm a black person and I'm usually the only black person in a project. Everybody else is Caucasian, European, there are no visible minorities involved in any of these patient and family advisor projects that I've been involved in which is a big concern to me*. (Participant A008)

This led some racialized participants to identify racism as something that needed to be immediately addressed in patient policy engagement activities, ‘We need to identify it when it [racism] happens and we need to attack it head‐on and address it and make sure that it's not part of the equation’, (Participant A008). Overall, participants who experienced racial discrimination highlighted the need for a higher level of cultural competence in policy engagement opportunities.

Gender bias was also mentioned by participants as a barrier to participation in policy, ‘I think when I was younger, let's say even up until about five or six years ago, one of the barriers was being female when dealing with male medical professionals, I know what was happening, a lot of it was cultural’, (Participant B006). While some participants who were part of the younger age demographic felt that other policy committee members did not value them equally due to their age. Furthermore, some participants stated that some of the languages used to label certain demographic age groups (e.g., labelling those aged 45–65 as older adults) could be disempowering to these individuals and may result in loss of input from this age group, ‘And it's just like – “well, that's the category we have for you so we're going to call you that anyway”,’ (Participant B005). The experiences of participants that highlighted these forms of discrimination emphasized the requirement for a higher level of inclusivity in the engagement of patients in policy development, implementation and evaluation.

**Table 2:** Additional barriers and facilitators to engagement at the policy level

Description	Barriers	Facilitators
Communication skills	Patient engagement depends on the patient's ability to share their experiences. Lack of communication skills may hinder patient engagement in policy.	Education and encouragement of patient partners to voice their needs.
Judgement and blacklisting	Patient partners may feel judged for their experiences and opinions. Patient partners may not feel comfortable sharing their opinions because they are afraid it will impact the quality of, and access to, care. ‘Many seniors worried about being blacklisted, worried about payback from the hospital or if they go in to the local doctor… you'll never get a doctor again’. (Participant O004)	Mutual respect for patient perspectives. Building trusting relationships.
Time constraints and compensation	Work/personal commitments. ‘I'm not getting any time off work to be a patient advocate, but it is literally a part‐time job’. (Participant O006) Lack of incentive, reimbursement or honorarium to involve diverse populations, such as working individuals, youth and individuals from marginalized communities. ‘Why don't we offer some pay to the people who can't afford and then the rest of us can still be volunteers… it's always been, like, ‘well no, we have to do it for everybody’. And then, of course, nobody has the budget to pay everybody, right?’. (Participant O002)	Short‐term commitments (1 or 2 meetings) for committees for patients who may not be able to be regularly involved as it may interfere with important personal needs (e.g., work, family, health).
Knowledge and awareness	Patients/caregivers not already connected to engagement opportunities do not learn about them. Lack of efforts to reach patient partners, ‘There wasn't any public announcements that I'm aware of that looked for general members of the general public’. (Participant O004)	Awareness that these positions do not require patients to have previous knowledge about the health care system and that their personal experiences are valuable. Advertising engagement opportunities in social media, newspapers and on television.

## 4 DISCUSSION

The objectives of the interviews were to understand if and in what ways patients, family members and caregivers were or wanted to be engaged in policy related to team‐based PHC. The key themes from the interview data offer important insights into areas of patient–partner engagement in PHC team policy development, implementation and evaluation that require improvement.

### 4.1 Tokenism and power imbalances

Many participants experienced barriers to their engagement in PHC policy even when they were given opportunities to be engaged in the policy process. Tokenism has been determined to be a significant hindrance to the engagement of patients in the policy process^4^, ^20^ and several of the participants confirmed this finding. Participants stated that tokenistic engagement diminished their ability to contribute as patients, family members and caregivers and left them feeling more like they were a box to check off. Participants described the importance of being engaged with other stakeholders, such as policymakers, decision‐makers and health providers throughout the whole decision‐making process. This emphasizes the need to adapt the spectrum of public participation (i.e., inform, consult, involve, collaborate and empower) outlined in the IAP2 framework^21^ to facilitate patient, family member and caregiver engagement in PHC policy activities. Increasing opportunities for patients, family members, and caregivers to become more engaged with PHC policy highlights the importance of creating new opportunities and giving patients, family members and caregivers diverse opportunities for full engagement in policy development, implementation and evaluation. Providing opportunities for genuine engagement and taking patients', family members' and caregivers' feedback into consideration could help the PHC system gain more diverse perspectives and become more effective at serving the needs of patients.

Multiple participants expressed dissatisfaction with the power imbalances that inhibited them from being equitable partners in PHC policy discussions. This result verifies previous statements in the literature stating that power imbalances often lead to health care professionals having a greater influence over policy than patients.^22^ Change is needed in the organizational culture of patient, family member and caregiver engagement in PHC policy development, implementation and evaluation (e.g., provincial policy committees, patient advisory groups/committees, national networks) to prevent and address such power imbalances in the future. Previous research regarding power imbalances in patient engagement activities suggests strategies, such as increasing the use of an ongoing communication style between health care professionals and patients, to encourage collaboration between the public and health care professionals.^23^ Promoting awareness and increasing health care professional's knowledge of patient engagement can shape how health care professionals value patient engagement.^24^ Creating programs like this can increase an understanding of the importance of patient engagement among health care professionals and could increase their willingness to treat patients as equitable partners.

### 4.2 Policy engagement opportunities

Many of the participants interviewed had been engaged in policy in some capacity, but there was an overall lack of awareness among participants regarding engagement opportunities in PHC policy development, implementation and evaluation. Likewise, individuals who were both engaged and not engaged in policy mentioned the lack of engagement opportunities available to them. These results reflect previous research findings that have reported the lack of opportunities for patients to become engaged in policy, and the lack of awareness of engagement opportunities among patients, family members and caregivers.^4^ This feedback highlights the need for patient partner organizations to discover innovative ways to advertise PHC policy engagement opportunities, potentially advertising in clinics, hospitals and other locations where patients are likely to see recruitment opportunities. Furthermore, innovative methods of recruitment that support the inclusion of patients from underrepresented demographic groups should be used; this could include methods, such as recruiting at locations where it is common for these groups to gather (e.g., drop‐in centres, faith‐based organizations) and recruiting through building trusting relationships with key members of Indigenous communities. These results also indicate a need for government and health organizations to create more PHC policy engagement opportunities by providing adequate funding and organizational support.

Participants also expressed experiences of racism, sexism and ageism, while attempting to engage in PHC policy processes and highlighted a lack of diversity among those engaged. Given that the purpose of engaging patients in policy is to obtain diverse perspectives on the health care system,^4^ the existing methods for engaging patients, family members and caregivers in PHC policy development, implementation and evaluation are inadequate to facilitate the voices of certain demographic groups (e.g., younger individuals, racialized individuals and individuals with low socioeconomic status) from being heard. Policymakers should encourage diversity in the recruitment of patients and facilitate the inclusion of a variety of perspectives to ensure decisions in the PHC system can be inclusive of all patients in the population. The discussion surrounding the lack of diverse patient representation in engagement opportunities has been noted by other scholars as an area requiring improvement.^4^, ^25^, ^26^ While it is promising to see that patients, family members and caregivers are being engaged, it is crucial for the PHC system to increase the number of patient partner positions and recruit individuals from diverse demographic groups moving forward.

In alignment with increasing diversity, the structure of patient engagement in PHC policy development, implementation and evaluation could be improved to increase the diversity of patient engagement participants by making opportunities more accessible. Many of the participants in this study who were engaged in the policy process were retired or on disability and very few were employed part or full‐time. Furthermore, participants stated that work commitments often were a barrier to participants' engagement in policy. This is consistent with other literature as meetings for patient engagement groups have been previously stated to often be scheduled during the workday, making it impossible for individuals who need to work to earn an income to participate.^25^ Many of the participants cited work commitments, the lack of transportation or technology, the inability to make large time commitments and their physical health conditions as barriers to their engagement in the policy development, implementation and evaluation. Steps should be taken to address these issues and needs. Other work in this area has recommended creating more institutional structures that facilitate patient engagement, such as covering transportation costs for patients, covering childcare or caregiver support, remunerating patients for the time taken off work, interpreter fees and covering other costs associated with engagement.^25^ Creating supports such as these may break down some of the barriers, which may be currently preventing those from underrepresented demographic groups from participating in the policy process.

### 4.3 Genuine patient engagement

Participants reported numerous continued barriers to patient engagement (e.g., tokenism, power imbalances, lack of awareness/opportunities, accessibility challenges and lack of diversity in patients recruited). These barriers are consistent with those that have been previously reported in the literature (for example^4^, ^20^, ^22^). Our results indicate that these barriers continue to persist in patient engagement despite the efforts in advancing patient engagement in policy. There are still instances where patients' contributions are not valued appropriately, and more effort must be put into providing patients with genuine engagement opportunities.

## 5 STRENGTHS AND LIMITATIONS

One of the strengths of this study is that interviews were conducted in three different provinces across Canada allowing perspectives from a diversity of locations within the country to be heard. Similarly, in each province, there was variation in the age and geographic location of participants, which can strengthen generalizability. The interview structure was designed to create a safe space for participants and allowed participants to share valuable insights on their engagement in PHC policy activities, barriers to engagement that they experienced and future ways to improve the structure of patient engagement. Finally, patients with and without experience in PHC policy were included in the study to gather the perspectives of those with different lived experiences in policy engagement.

The majority of participants recruited were females and White. This is important to acknowledge as those of differing sex and ethnicity may have different lived experiences when utilizing the health care system and being engaged in PHC policy. Therefore, although the experiences of the participants provide rich information and insight into patient engagement in PHC policy, they may not be reflective of the overall population in BC, AB and ON.

One limitation of interpretative description is that the context of previous knowledge used in interpretative description is dependent on the data obtained from the literature review performed.^10^ To ensure that a robust literature review was conducted, researchers developed comprehensive search criteria and reviewed relevant literature from each province. Furthermore, it has also been stated that researchers using interpretative description may focus on certain accounts in the data that are more compelling to them, due to their interactions with participants during the interview phase.^10^ To account for this, there were multiple researchers who conducted the interview analysis.

Finally, the research team considered their own personal and professional experiences potentially influencing their interpretation of the results. The research team made a conscious effort to outline their potential biases. The data underwent multiple rounds of review by all members of the research team. Furthermore, a patient partner involved in the project (J. B.) reviewed the results of the study and made significant contributions to the preparation of the manuscript.

## 6 CONCLUSION

Overall, the patient interviews revealed some important progress in the engagement of patients, family members and caregivers in policy development, implementation and evaluation in the PHC systems of BC, AB and ON. The experiences of participants indicated that although many of the participants were engaged in PHC policy as patient partners, many participants expressed concerns regarding the lack of patient engagement opportunities and mentioned there were significant barriers hindering genuine patient engagement. Participants also shared important information regarding their experiences with PHC teams, which provided insight into their motivation to be engaged in PHC policy. Moving forward, it will be critical for stakeholders to consider the key themes related to motivation for policy engagement, experiences in policy engagement and barriers to engagement in policy. These themes should be considered to increase patient, family member and caregiver engagement in PHC policy development, implementation and evaluation and improve the integration of care provided to patients through PHC teams.

## AUTHOR CONTRIBUTIONS

Peter Hirschkorn: *Data collection in British Columbia (BC); data analysis and interpretation in BC, Alberta (AB), and Ontario (ON); drafting the article; critical revision of the article and final approval of the version to be submitted*. Ashmita Rai: *Data collection in BC, data analysis and interpretation in BC, AB and ON; drafting of the article; critical revision of the article and final approval of the version to be submitted*. Simone Parniak: *Data collection in ON; data analysis and interpretation in ON; critical revision of the article and final approval of the version to be submitted*. Caillie Pritchard: *Data collection in AB, data analysis and interpretation in AB, critical revision of the article and final approval of the version to be submitted*. Judy Birdsell: *Conception or design of the work, critical revision of the article, and final approval of the version to be submitted*. Stephanie Montesanti: *Conception or design of the work, data analysis and interpretation in AB, critical revision of the article, and final approval of the version to be submitted*. Sharon Johnston: *Conception or design of the work, data analysis and interpretation in ON, critical revision of the article, and final approval of the version to be submitted*. Catherine Donnelly: *Conception or design of the work, data analysis and interpretation in ON, critical revision of the article, and final approval of the version to be submitted*. Nelly D. Oelke: *Conception or design of the work, data analysis and interpretation in BC, AB and ON, critical revision of the article, and final approval of the version to be submitted*.

## CONFLICTS OF INTEREST

The authors declare no conflicts of interest.

## Supporting information

Supplementary information.

## Data Availability

Data are not freely available as per ethics protocol.
