# Supplementary material for: Patient, family member and caregiver engagement in shaping policy for primary health care teams in three Canadian Provinces
Source: Health Expect. 2022 Jun 15;25(4):1730–40. doi: 10.1111/hex.13516 (PMC9327874; doi:10.1111/hex.13516)
Supplement: Supplementary file 1 — Supplementary information. [file HEX-25--s001.docx]

**Appendix A: Interview Guide for Patient Interviews**

**Primary Health Care Teams Policy Project**

**Interview Guide**

| Questions | Probes |
| --- | --- |
| 1. Can you tell me about your experiences as a patient or caregiver with primary health care teams (two or more health care providers working together to provide care in the community)? | - What professions were involved? (e.g., physician, nurse, social worker) - What types of care did you receive? (e.g., PHC, preventive care, annual check-up) - On a scale of 0-10, where 0 being not good and 10 being excellent, how would you rate your overall experience? |
| 1. Did you feel like you were a member of the team that was working to plan your/ your significant other’s care? | - How were you involved? E.g., in receiving information, decision-making about care - As a patient or caregiver, what do you see as the essential elements which need to be included in an integrated primary health care team? E.g., What, for you, would define whether or not you are receiving integrated primary health care? |
| 1. Can you comment on how well the services you needed or received were connected or linked together? | - Seamless care, continuous care (How was the care? Continuous or fragmented?) - Communication (How did the various health care providers who cared for you communicate with each other?) - Working together (What did you observe that led you to say they worked well together, or they did not work together?) - Common goals for the patient (Did you feel that all the care providers who cared for you had the same goals for you or your loved ones?) |
| *Now we are going to switch a bit of focus. I am going to ask you questions that relate to your role as someone who influences the guiding principles or courses of action (that may or may not be related to your personal care or that of your family member or friend) and then ask you questions about your overall participation or involvement (your involvement could include: providing your opinion, participating as a member of a committee, asking others for their input, involvement as a representative of another group who collaborates with primary care teams).* |  |

| 1. Have you ever participated in developing, applying or evaluating policies/guiding principles that would affect care received by the patients? | - Locally - Regionally - Provincially - Nationally |
| --- | --- |
| 1. Have you had any involvement with your family doctor’s clinic/ primary health care team that was not directly related to your own health care or that of your loved one? | - Committee involvement (e.g., development of services) - Research initiatives (e.g., designed surveys, participated in interviews, or been a part of research team) - Other types of activities |
| 1. Have you ever been asked for your opinion on how to improve primary health care services? | - How did you feel about your voice being heard? - Did any practice or procedure change as a result of the activity you were involved with? - If not, what would have helped to have your voice/perspectives heard? |
| 1. If you haven’t been involved previously, would you like to be involved in such activities? How would you like to be involved? | - Involvement in policy development, implementation, and evaluation Different types of activities for different levels of involvement - Different types of activities for local, regional, provincial and national involvement |
| 1. What were the barriers or challenges that you have come across while trying to participate more? |  |
| 1. Do you have anything else you would like to share about any of the topics or another related topic? |  |
